# Bilaterian-like promoters in the highly compact *Amphimedon queenslandica* genome

**DOI:** 10.1038/srep22496

**Published:** 2016-03-02

**Authors:** Selene L. Fernandez-Valverde, Bernard M. Degnan

**Affiliations:** 1 School of Biological Sciences, The University of Queensland, Brisbane 4072, Australia

## Abstract

The regulatory systems underlying animal development must have evolved prior to the emergence of eumetazoans (cnidarians and bilaterians). Although representatives of earlier-branching animals – sponges ctenophores and placozoans – possess most of the developmental transcription factor families present in eumetazoans, the DNA regulatory elements that these transcription factors target remain uncharted. Here we characterise the core promoter sequences, U1 snRNP-binding sites (5′ splice sites; 5′SSs) and polyadenylation sites (PASs) in the sponge *Amphimedon queenslandica*. Similar to unicellular opisthokonts, *Amphimedon’s* genes are tightly packed in the genome and have small introns. In contrast, its genes possess metazoan-like core promoters populated with binding motifs previously deemed to be specific to vertebrates, including Nrf-1 and Krüppel-like elements. Also as in vertebrates, *Amphimedon*’s PASs and 5′SSs are depleted downstream and upstream of transcription start sites, respectively, consistent with non-elongating transcripts being short-lived; PASs and 5′SSs are more evenly distributed in bidirectional promoters in *Amphimedon*. The presence of bilaterian-like regulatory DNAs in sponges is consistent with these being early and essential innovations of the metazoan gene regulatory repertoire.

Spatiotemporal and cell type-specific gene regulation is prerequisite to multicellularity. Thus, any insights into the origin and evolution of animals requires a detailed understanding of gene structure – both regulatory and coding sequence – and the interplay between available transcription and regulatory factors with regulatory sequences that control gene expression^1^,^2^,^3^,^4^. Although the evolution of metazoan transcription factor families has been relatively well documented^5^,^6^,^7^,^8^,^9^, the evolution of regulatory sequences and gene regulatory networks remain restricted to a handful of case studies in bilaterian arthropods, chordates, nematodes and echinoderms^10^,^11^,^12^,^13^,^14^,^15^,^16^. It currently remains an open question as to what extent early-branching, morphologically simple animals, sponges (poriferans), ctenophores, placozoans and cnidarians, share regulatory elements with more complex bilaterians.

Although genomic regions that regulate gene expression often lie at a distance from the transcription start site (TSS)^17^, sequences overlapping with and in the vicinity of the TSS – core or basal promoter elements – are necessary for the integration of *cis*-regulatory inputs and the initiation of transcription^18^,^19^,^20^. These promoters also contribute directly to the regulation of gene expression in development and cell differentiation beyond simply the coordination of transcription^21^,^22^,^23^.

Some core promoter regulatory elements appear to be broadly conserved amongst eukaryotes (e.g. the TATA-box^24^,^25^), although there is marked variation in promoters between genes within a given species^18^,^26^,^27^,^28^. For instance, TATA-boxes and the initiator elements (Inr), which are considered the only core promoter motifs conserved from yeast to humans^25^, are present in only ~25% of human promoters^25^. Further, comparative analyses^2^,^25^,^29^,^30^ reveal that while there are a handful of other conserved elements that are present in some animal promoters [e.g., the transcription factor IIB recognition element (BRE), downstream core element (DCE), downstream promoter element (DPE), DNA recognition element (DRE) and motif ten element (MTE)], many regulatory sequence motifs appear to be restricted to specific species^3^,^26^,^31^,^32^. The diversity of promoter classes within and between metazoan species supports the view that this region contributes to the complex regulation of gene transcription in metazoans^3^,^33^.

The increase in the number and diversity of draft metazoan and eukaryotic genomes provides an opportunity to understand the relationship between the regulatory capacity of the genome and morphological evolution, including the origin of animal multicellularity. Leveraging on an increasingly detailed understanding of genome structure and function in model species, we use the reannotated draft genome of the demosponge *Amphimedon queenslandica*^34^, to begin to understand the relationship between gene regulation and animal evolution. Our findings are consistent with metazoan core promoter(s) originating in the pre-Cambrian before the divergence of eumetazoan and sponge lineages.

## Results

### The *Amphimedon* genome is compact

**Figure 1:**
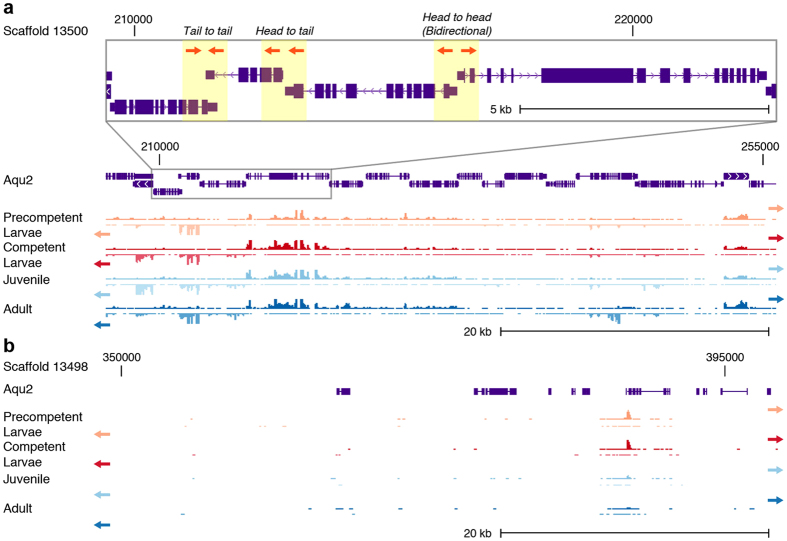
Examples of gene dense and depleted regions in the *Amphimedon queenslandica* genome. (**a**) A gene rich region with Aqu2 gene models shown in purple with thick lines depicting exons, mid size lines UTRs and thin lines introns. The wiggle tracks below show the RNA-seq expression of the Watson (top) and Crick (bottom) strand in precompetent larvae, competent larvae, juvenile and adult samples. The zoomed in region shows examples of genes orientated tail to tail, head to tail and head to head, with the direction of transcription shown by orange arrows and in introns as small arrow heads. (**b**) A gene depleted region. Color schema as in panel (**a**).

The reannotated *Amphimedon* genome contains 40,122 coding sequence gene models (excluding isoforms), covering nearly 65% of total genomic sequence^34^. These new gene models include better-annotated 5′ and 3′ untranslated regions (UTRs), allowing for the identification of transcript start and termination sites (Fig. 1). This new annotation has revealed the *Amphimedon* genome is phenomenally compact, having a median intergenic distance of a mere 587 bp^34^ and few gene deserts.

**Figure 2:**
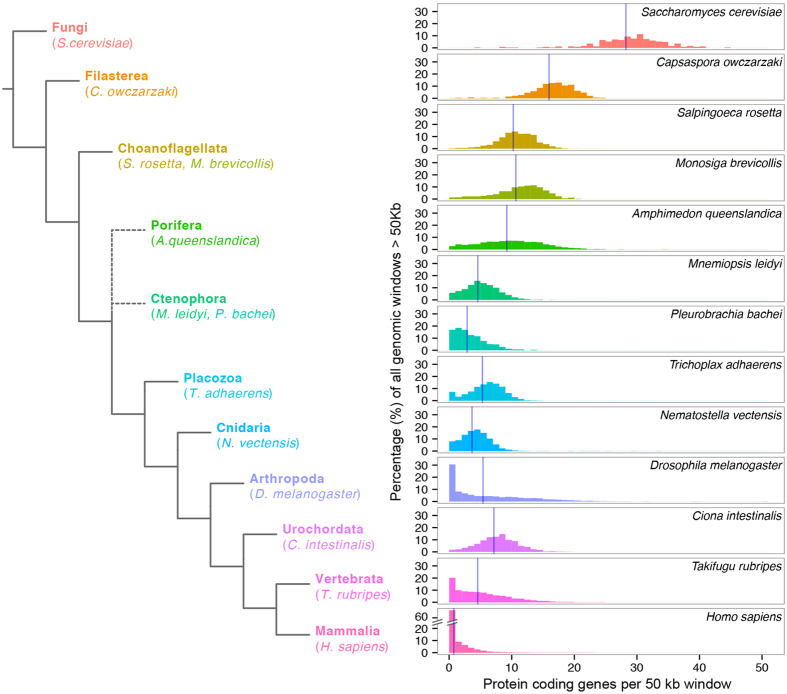
Comparative analysis of gene density in the genomes of representative opisthokonts. This analysis includes four non-metazoan opisthokonts (*Saccharomyces cerevisiae, Capsaspora owczarzaki*, and two choanoflagellates, *Salpingoeca rosetta* and *Monosiga brevicollis*), the sponge *Amphimedon queenslandica*, two ctenophores (*Mnemiopsis leidyi* and *Pleurobrachia bachei*), the placozoan *Trichoplax adhaerens*, the cnidarian *Nematostella vectensis*, the arthropod *Drosophila melanogaster*, and three chordates (*Ciona intestinalis, Takifugu rubripes* and *Homo sapiens*). Their relationships are shown to the left and the gene density distribution for each species is shown to the right. The percentage of genomic windows (y-axis) by the number of protein coding genes per 50 kb genomic window (x-axis) is shown, using data from all genomic scaffolds longer than 50 kb. Vertical blue lines in each panel mark the average gene density (see also Table 1). To diminish bias caused by multiple gene isoforms in well-characterized genomes (i.e. *D. melanogaster*), genes were counted as occurrences of a uniquely annotated genic 5′ ends (see Table 1). The broken axis for *H. sapiens* is to avoid scale distortion in other species.

To place the gene density of the *Amphimedon* genome within a comparative framework, we surveyed the number of protein-coding genes in non-overlapping 50 kb windows of genomic DNA in a range of animals, two choanoflagellates, a filasterean and yeast (Fig. 2, Table 1). This survey of animal genomes is not exhaustive but includes representatives of ctenophores, placozoans and cnidarians, along with bilaterians with comparable genome sizes or known to have relatively compact genomes within the taxon they represent (e.g. the pufferfish, *Takifugu rubripes*). We also included the human genome in this analysis.

We find that *A. queenslandica* has one of the most gene dense metazoan genome currently known, with a median of 9 genes per 50 kb. Only 2.7% of the *Amphimedon* genome is depleted of genes (Fig. 2, Table 1). The gene density of *Amphimedon* resembles that of the choanoflagellates *Salpingoeca rosetta* and *Monosiga brevicollis*, which have a median gene density of 10 and 11 per 50 kb, respectively, although it is less dense than the more evolutionary distant filasterean *Capsaspora owczarzaki*, with a median of 16 genes per 50 kb (Fig. 2, Table 1).

Gene density differences cannot be solely attributed to differences in genome size. The *A. queenslandica* genome (166.7 Mb) is over three and four times the size of that of *S. rosetta* (55.4 Mb) and *M. brevicollis* (41.6 Mb), respectively (Table 1). Although both the ctenophore genomes are similar in size to the *Amphimedon* genome (*Mnemiopsis leidyi*, 155.9 Mb; *Pleurobrachia bachei*, 156.1 Mb), they have a markedly lower median gene density (5 and 2 genes per 50 kb, respectively) and a higher percentage of gene depleted regions (5.8 and 17% in *M. leidyi* and *P.bachei*) (Table 1). These values for the ctenophores are closer to those found in *Nematostella vectensis* (356.6 Mb), although the cnidarian genome is nearly twice as large (Fig. 2, Table 1). The urochordate *Ciona intestinalis* has a smaller genome than *A. queenslandica* (115.2 Mb) and only 1.7% of its genome is composed of gene-depleted regions (Fig. 2), yet it is still less gene dense than that of *A. queenslandica*, with a median of seven genes per 50 kb (Fig. 2). Even the miniature genome of the holoplanktonic urochordate *Oikopleura dioica* (65 Mb) has a gene density similar to *Amphimedon*^35^. The genomes of *Drosophila melanogaster, Takifugu rubripes* and *Homo sapiens* are the least gene dense, and a mosaic of gene dense and depleted regions (Fig. 2, Table 1).

The *A. queenslandica* genome contains 18,054 (45%) genes that have the potential to be transcribed in opposite directions off the same core promoter. 11,379 of these head-to-head genes have transcription start sites (TSSs) 1 kb or less away from each other, and thus may be under the control of a bidirectional promoter (Table 2), as previously defined^36^,^37^. This makes *Amphimedon* currently the animal with the highest number of potential bidirectional promoters; 4,398 *D. melanogaster* genes (32.2%) are transcribed from bidirectional promoters^36^.

### *Amphimedon* promoters are enriched in elements present in bilaterian promoters

Using deep stranded expression data from a variety of developmental stages^34^, we identified 3,309 gene models (8.2% of total) with both RNA-seq supported 5′ gene-ends (annotated as described in^34^) and a promoter that did not overlap with another gene promoter (Supplementary Table 1). This is comprised of 330 bidirectional (when both head-to-head promoters have 5′ ends), 645 putative bidirectional (when one head-to-head promoter pair has a single gene with an annotated 5′ end) and 2,334 unidirectional (when no evidence of bidirectional or divergent transcription is found within 1.0 kb of the identified promoter) promoters. Analysis of the nucleotide composition in the vicinity of the TSSs of these three promoter types (Supplementary Fig. 1) reveals no sequence differences, with all having an increase in C and G nucleotide frequency at and just after the TSS (Supplementary Fig. 1).

**Figure 3:**
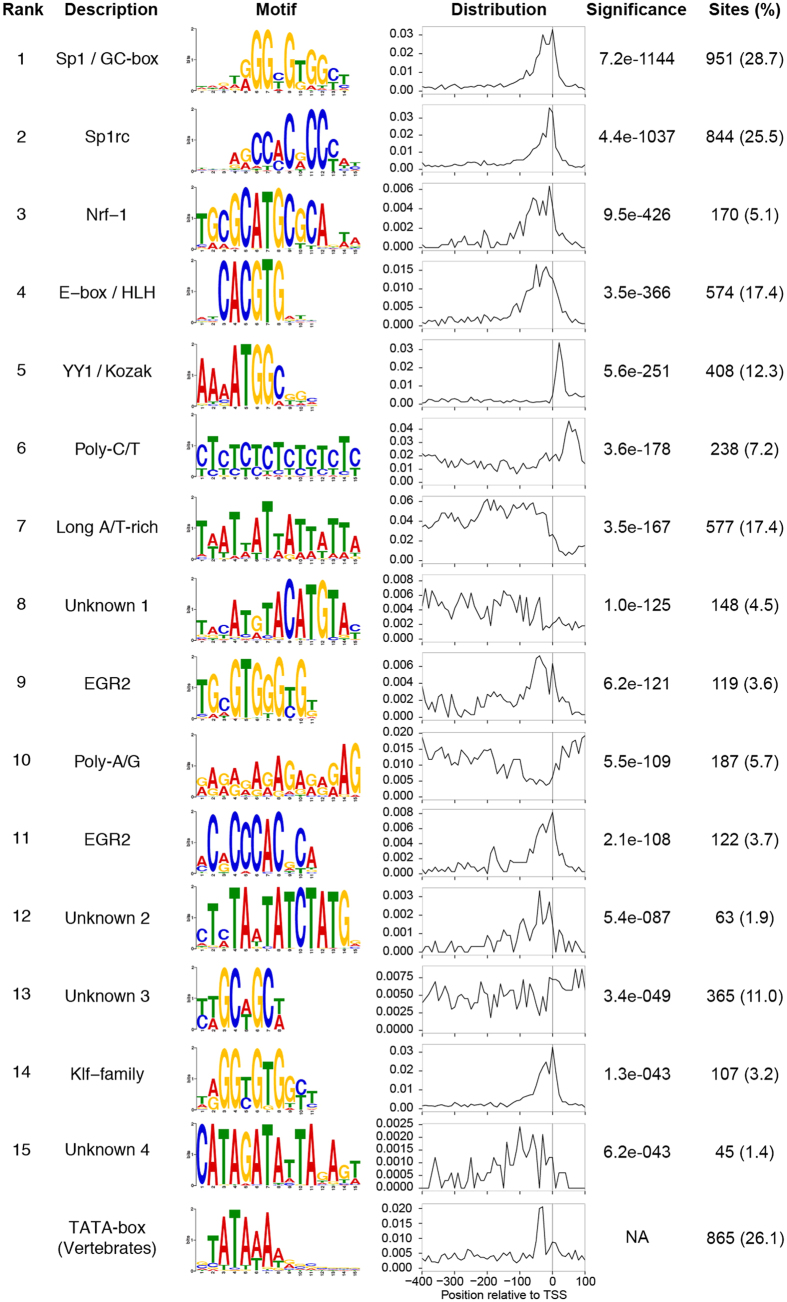
DNA motifs overrepresented in Amphimedon queenslandica promoters. The sequence logo of DNA motifs significantly enriched in the promoters and the TATA-box are shown. The frequency per 10 bp bin from −400 bp to +100 bp relative to the TSS (grey vertical line) is displayed in column 4. The significance value is the e-value of the log likelihood ratio of each motif. The right column shows the number of promoters, amongst the 3,309 promoters surveyed, and the percentage of promoters (in parentheses) that possess the motif.

An unbiased survey of the DNA sequences most overrepresented in the vicinity of the TSSs of the 3,309 coding gene representatives revealed 15 motifs significantly enriched in unidirectional, bidirectional and putative bidirectional promoters (Fig. 3; Supplementary Table 2). The most abundant element is a specificity protein 1 (Sp1)-like GC–box, whose frequency is enriched within 50 bp upstream of the TSS peaking right at the TSS (Fig. 3). This GC-rich motif is more prevalent in the bidirectional and putative bidirectional promoters (~36%) compared to unidirectional promoters (29%) (Supplementary Table 2). The reverse compliment of this motif (Motif 2, Fig. 3) is also overrepresented in promoter regions (coding strand) and is most common at the TSS (Fig. 3). This motif is also more abundant in putative bidirectional and bidirectional promoters (Supplementary Table 2). E-box sequences are enriched around the *A. queenslandica* TSSs and practically identical to the known canonical sequence of a special type of E-box known as a G-box, characteristic of plants^38^. This motif is four times more common in bidirectional and putative bidirectional promoters than in unidirectional promoters (Supplementary Table 2). A motif containing the Kozak sequence (translation initiation site) is also overrepresented in *A. queenslandica* promoters, although only in a fraction of the genes (Fig. 3). This motif is AT rich, similar to previously reported Kozak consensus in cnidarians and yeast^39^. This motif also bears close resemblance to the YY1 active motif in immortalized human cell lines^40^. The YY1/Kozak motif is more prevalent ~10 bp after the transcription start site, although its low abundance suggests that some genes begin translation further than 100 bp downstream of the annotated TSS (Fig. 3). The post-TSS position of the identified Kozak sequence confirms the use of only genes with high confidence 5′ UTRs and a strict motif identification approach to identify promoter elements.

Additional motifs characteristic of bilaterian promoters include Nrf-1, EGR-2 and KLF family binding sites (Fig. 3). The Nrf-1 and EGR-2 binding sequences are distributed close to the TSS but without a clear positional association (Fig. 3). The KLF-family motif, when present, is found at a similar distance to the TSS as the Sp1/GC-box motif. This is because of the sequence similarity between the two motifs (Fig. 3). Poly-CT patches are enriched more downstream of the TSS site. Four additional motifs that have no clear similarity with previously identified binding motifs are also significantly enriched around the TSS; only one of them, “Unknown 2”, is more prevalent close to the TSS than the surrounding regions (Fig. 3).

Many of the promoter elements that we identified in the survey of 3,309 genes with well-defined TSSs and promoters are also enriched in the putative promoters in other protein-coding genes in the *Amphimedon* genome, despite these genes having less resolved TSSs (Supplementary Fig. 2). These enrichments include Sp1/GC-box and the Sp1rc motifs, which are the most abundant elements that precede or overlay TSSs across the genome.

Among the most highly enriched motifs in the *Amphimedon* promoters surveyed, we found only one A/T-rich motif, with similar nucleotide composition to the TATA-box. Nevertheless, this motif had no specific positional enrichment and was prevalent across the whole region upstream of the TSS (Fig. 3). Scanning the promoters using the TATA-box consensus from vertebrates, we find that the TATA-box frequency peaks approximately 30 bp upstream of the TSS as expected^33^. Similar to previous reports in other organisms^25^,^41^, we find that (865) 26% of all surveyed promoters contain an identifiable TATA-box, regardless of promoter type (Fig. 3). This is perhaps not surprising given the *Amphimedon* genome is also relatively AT-rich (64.2%) compared to many other animal genomes (Table 1).

Other animal core promoter elements, including Inr, BREu, BREd, DPE, MTE, CEBP, and XCPE1, were not identified as enriched in our analysis. A survey for these motifs shows the BREu is most abundant in the region corresponding to the TSS (Supplementary Fig. 3), and closely resembles the distribution of the Sp1/GC-box motif on the promoter region (Fig. 3). This observation, in addition to the similarity between BREu and the Sp1/GC-box motif, suggest that the latter element might be acting as BREu in *A. queenslandica*. The metazoan-specific ETS motif is infrequent in *Amphimedon* promoters but localizes to the region around the TSS when present (Supplementary Fig. 3). Other conserved motifs, including the human and *Drosophila* Inr, BREd, DPE, CEBP and XCPE1 motifs, were either absent, present in very low frequencies or appear to be randomly distribution in the core promote region, possibly reflecting the inherent low specificity of these motifs^42^ (Supplementary Fig. 3).

### Differential enrichment of poly(A) signals upstream and U1 snRNP-binding sites (5′ splice sites) downstream of *Amphimedon* core promoters

**Figure 4:**
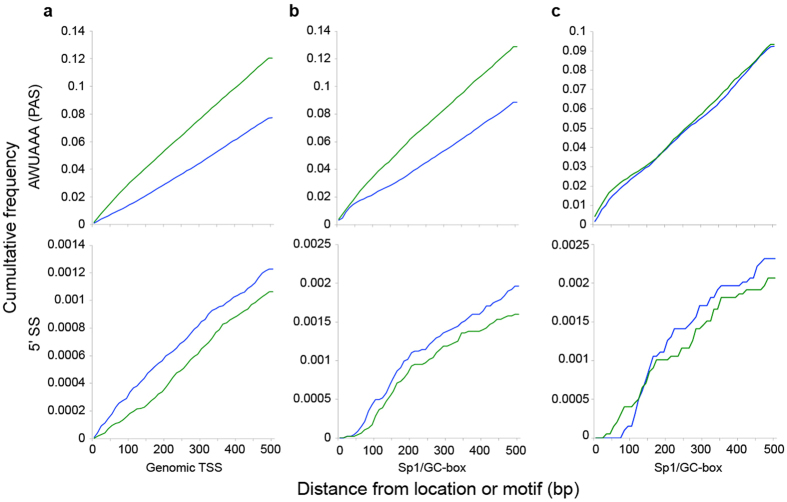
Cumulative frequency of polyadenylation and U1 snRNP-binding sites (5′ splice sites) sequences upstream and downstream of *Amphimedon* TSSs. The polyadenylation signal (AWUAAA PAS) and the U1 snRNP-binding sites (5′ splice sites; 5′SS) frequencies are shown in top and bottom panels, respectively. Blue lines, cumulative frequency for 500 bp downstream of the TSS in the sense direction. Green lines, cumulative frequency for 500 bp upstream in the antisense direction. (**a**) All TSSs in the genome. (**b**) Promoters with an identifiable Sp1/GC-box motif (**c**) Bidirectional promoters with an overlapping pair of Sp1/GC-box and Sp1rc motifs.

Vertebrate transcriptional elongation is driven by pervasive bidirectional and divergent transcription from promoters, followed by quick destabilization and degradation of unstable transcripts from the non-coding strand^43^,^44^. The enrichment of U1 snRNP-binding sites (5′ splice sites; 5′SSs) and poly(A) signals (PASs) downstream and upstream of the TSS, respectively, correlate with transcript stability^45^. A survey of the abundance of PAS and 5′SS motifs in relation to all TSSs in the *Amphimedon* genome reveals PAS motifs accumulate more rapidly in the antisense (non-coding) than in the sense direction (coding) and 5′SS motifs are more prevalent on the coding strand, albeit at much lower frequency (Fig. 4a). A similar accumulation pattern is observed when the survey is restricted to genes with core promoters containing Sp1/GC box or Sp1rc motifs (Fig. 4b). 5′SS motifs accumulate nearly twice as rapidly downstream of these highly prevalent motifs compared to TSSs across the genome (Fig. 4a,b).

Analysis of bidirectional promoters with co-occurring and/or overlapping Sp1/GC-box and Sp1rc motifs revealed PAS accumulated at almost identical rates in both sense and antisense direction (Fig. 4c). These motifs are over three times as likely to co-occur in a promoter than expected by chance (Supplementary Table 3, see supplementary note). 5′SS sequences tended to accumulate more rapidly in the sense than the antisense strand. Although the 5′SS cumulative frequency rises at almost similar rates between strands, they are not as similar as that of the PAS signal. This difference in cumulative frequency suggests polyadenylation is a more prominent transcriptional regulatory element than splicing in *Amphimedon*.

## Discussion

*Amphimedon queenslandica* has one of the most gene dense genomes in the animal kingdom, with a median of 9 genes per 50 kb and a median intergenic distance of 0.59 kb. *Amphimedon*’s genome organization, average intron size and alternative splicing patterns^34^,^46^ are more similar to unicellular holozoans than to other animals. These may be symplesiomorphic traits that are shared between sponges and unicellular holozoans, with other metazoan lineages evolving larger introns and intergenic regions. Alternatively *Amphimedon’s* high gene density may be the result of a secondary reduction in intergenic and intron DNA size from a last common metazoan ancestor that had already had an increased amount of non-coding DNA. Secondary increases in gene density appear to have occurred elsewhere in the animal kingdom (e.g. urochordates).

The enlargement of intergenic space increases the availability of non-coding, and presumably sequences that are less constrained to evolve into transcription factor target sites^47^,^48^. Although most developmental transcription factor families evolved early in metazoan evolution, prior to the divergence of extant phyla^5^,^8^, a majority of these families underwent further expansion and diversification early in eumetazoan evolution after this lineage diverged from poriferan and ctenophore lineages. The increase in transcription factor family membership in eumetazoans is likely to have led to an expansion in regulatory potential and gene regulatory network complexity and may underlie the difference in morphological evolution in sponges and eumetazoans^5^. The sponge body plan has remained unchanged since before the Cambrian^49^, while the eumetazoan lineage consists of a huge diversity of body plans. Ctenophores, whose transcription factor repertoires are similar to sponges^50^,^51^, have larger intergenic regions than *Amphimedon* and are considered morphologically more complex.

The high gene density of the *Amphimedon* genome results in a high frequency of small intergenic regions, overlapping transcription start and termination sites, and bidirectional promoters. Almost one third of *A. queenslandica* genes appear to be transcribed from a bidirectional promoter. We did not detect any major differences in nucleotide composition or abundance or distribution of binding motifs between unidirectional and bidirectional core promoter regions. Despite the relative AT-richness of the *Amphimedon* genome, there is a marked increase in the frequency of CpG dinucleotides at the *Amphimedon* TSS, similar to mammalian promoters^52^. CpG dinucleotides are highly mutable and rapidly lost in organisms with active DNA methylation mechanisms, as previously proposed for *Amphimedon*^52^,^53^,^54^. The increase in CpG around the *Amphimedon* TSS suggests that the mechanism responsible for maintaining low methylation levels of CpG dinucleotides in core promoters may have evolved prior to the divergence of poriferans and eumetazoans, or independently in sponges and vertebrates.

### Ancient origin of animal promoters

Several of the most prevalent sequence motifs are overrepresented in and around the *Amphimedon* promoter include sequences previously found in bilaterians or specifically vertebrates, including KLF, Nrf-1 and ERG binding sites. The most prevalent among these is the Sp1/GC-box like motif, present in about 29% of all promoters. Given its prevalence and similarity to bilaterian BREu elements^3^, the Sp1/GC-box may play a similar function in core promoters in this sponge. The increased presence of Sp1/GC-box and Sp1rc motifs in *Amphimedon* bidirectional promoters is consistent with these elements promoting transcription in both directions.

It is worth noting that no other conserved bilaterian transcription initiation motif is enriched in *A. queenslandica* promoters. However, a deliberate search for conserved promoter motifs did reveal a relatively well-preserved TATA-box motif 30 bp upstream to the TSS in about 26% of the *Amphimedon* genes. Its location relative to the TSS is in agreement with the known TATA distances in other metazoans^33^.

Members of the Sp/KLF family of proteins are known to bind to either GC-box^55^ or CACC-box motifs^56^, both which share similarity with Sp1rc, the second most abundant sequence enriched in *Amphimedon* promoters. This protein family, characterized by a three C2H2 zinc finger DNA-binding domain at the C-terminus^55^, arose and expanded early in metazoan evolution^57^,^58^,^59^. *Amphimedon* has about ten Sp/KLF family members (Fernandez-Valverde *et al*. unpublished^59^). The Sp transcription factor family is also known to help recruit TFIID (part of the pre-initiation complex) and GATA1 to the transcription complex^60^,^61^,^62^. Sp1 has also been shown to activate transcription without enhancing the DNA binding activity of the TATA box-factor^63^. The high abundance of the GC-box motif, which was also previously identified as enriched in the promoter region of *A. queenslandica* ribosomal proteins^64^, is consistent with Sp/KLF proteins being major recruiters of TFIID to the TSS in this sponge.

The Nrf-1 transcription factor gene family appears also to be restricted to metazoans. The *Amphimedon* genome has at least one member of this gene family. In addition to this study, the Nrf-1 binding motif has been found to be enriched in *X. tropicalis* promoters^26^ and in sponge ribosomal gene promoters^64^. Furthermore, a recent ultra-deep survey of transcribed enhancers in human immune cells displays almost a 100% similarity to the consensus sequence found in *Amphimedon*^65^. Together these observations suggest that Nrf-1 and its target binding site evolved before the divergence of sponge and eumetazoan lineages.

The poly-CT motif present in many *A. queenslandica* promoters has been previously identified in ribosomal gene promoters of this sponge, and in *N. vectensis* and *Trichoplax adhaerens*^64^. Although this motif has been proposed to be a metazoan innovation^64^, the presence of pyrimidine- and CTT-rich motifs in plant promoters^66^,^67^ suggests that the poly-CT motif present in sponge promoters may have a more ancient origin and possibly has been lost early in bilaterian evolution. Likewise, a plant-like E-box motif is also prevalent in *Amphimedon* promoters, suggesting it may be a conserved ancient feature of gene regulation.

### A conserved metazoan U1-PAS axis

It has been proposed that the perceived directionality of transcription downstream of active promoters arises from the rapid degradation of transcripts produced in the other direction from the non-elongating strand^43^,^44^. The differential accumulation of PAS and 5′SS sites in both directions away from promoters appears to dictate transcript stability, with PAS and 5′SS sites depleted and enriched, respectively, in the direction that yields stable transcripts.

*Amphimedon* displays a depletion of PASs and accumulation of 5 ′SSs in the direction of stable transcription, suggesting these mechanisms also contribute to transcription directionality in this sponge. *Amphimedon* has a high abundance of bidirectional promoters. In contrast to unidirectional promoters, PASs accumulate at almost identical rates both upstream and downstream of bidirectional promoters, consistent with the differential accumulation of PAS sites contributing to transcript stability in *Amphimedon*. The differential presence of PAS sites upstream and downstream of unidirectional and bidirectional promoters with Sp1/GC-box and overlapping Sp1/GC-box and Sp1rc motifs suggests promoters harboring these motifs are capable of triggering stable bidirectional transcription in *Amphimedon.*

Our findings are consistent with pervasive bidirectional transcription followed by quick destabilization of the non-elongating strand via mechanisms associated with the “U1-PAS Axis” emerged prior to the divergence of sponges and eumetazoans, and antedates the expansion of non-coding DNA in eumetazoans. Interestingly, the “U1-PAS Axis” also has been proposed as a mechanism for the origin of new genes^45^ as well as a crucial contributor to the emergence of stable non-coding RNA transcripts^68^,^69^. The apparent existence of this axis in *Amphimedon*, along with a long non-coding RNA repertoire similar to that found in bilaterians^70^, suggests that this mode of gene origination may be also an ancient feature of metazoan genomes.

## Summary

Overall, our observations are consistent with sponges having maintained genomic features that emerged early in metazoan evolution. The apparent dual unicellular-multicellular nature of the *Amphimedon* genome suggests that last common ancestor of sponges and eumetazoans had already evolved a regulatory core promoter to allow for context- and cell type-specific gene expression, yet had not evolved the ramified regulatory architecture observed in complex bilaterians. These evolved along the eumetazoan stem along with the expansion of key developmental transcription factor families^5^,^8^.

## Material and Methods

### Gene density analysis

The genomes and gene annotations of *H. sapiens, T. rubripes, C. intestinalis, D. melanogaster* and *S. cerevisiae* were downloaded through the ftp site of Ensembl (http://www.ensembl.org/info/data/ftp/index.html) on March 2014. Non-coding genes were filtered out using the “coding” biotype as specified by Ensembl. The genomes of *M. brevicollis, S. rosetta* and *C. owczarzaki* were downloaded from the Broad genomes “Origins of Multicellularity” website (http://www.broadinstitute.org/annotation/genome/multicellularity_project/Downloads.html) on December 2014. The genome of *T. adhaerens* was downloaded from the JGI website (http://genome.jgi.doe.gov/Triad1/Triad1.download.ftp.html) on December 2015. The NCBI repository was used to retrieve the genomes of *M. leidyi* (http://www.ncbi.nlm.nih.gov/nuccore/AGCP00000000.1/) *P. bachei* (http://www.ncbi.nlm.nih.gov/nuccore/AVPN00000000.1/) on May 2014. The *A. queenslandica* genome used in this study corresponds to that described in^71^.

All genomes were filtered to remove mitochondrial sequences and scaffolds shorter than 50 kb using awk. The gtf gene annotation files were converted to bed files using the UCSC utilities^72^. All TSSs were extracted and, if duplicated, collapsed into unique TSSs using a custom awk script. All genomes were divided onto non-overlapping windows of 50 kb and the number of TSSs overlapping each window were quantified using bedtools coverageBed^73^.

### Promoter identification

Promoter regions were identified using a custom perl script and a combination of the UCSC utilities^72^ and bedtools^73^. Briefly, using the TSS defined as the start position of the 5′ UTR we extracted 150 bp upstream of the core promoter and 50 bp downstream. All sequences that fell outside of an annotated genome scaffold or overlapped a gap in the scaffold were excluded. Promoters that had any overlap with another promoter were also excluded. To further refine our promoter set we only used promoters that were found on scaffolds longer than 10 kb that did not have long CpG islands (longer than 10 kb) and might be due to bacterial contamination. To identify bidirectional promoters, all promoters that were within 1 kb of each other and had evidence of bidirectional transcription were retrieved via a MySQL query of the *Amphimedon* genome UCSC database. To classify promoters into unidirectional, bidirectional and putatively bidirectional all core promoters were intersected with bidirectional intergenic regions of 1 kb or less using overlapSelect from UCSC utilities^72^. When two divergent core promoters overlapped a bidirectional intergenic region these promoters were considered “bidirectional”, while if only a single promoter overlapped the region it was classified as “putatively bidirectional”, as there was evidence of bidirectional transcription but only one of the genes had an annotated 5′ UTR and thus an annotated promoter. Promoters that did not overlap with these regions were deemed “unidirectional”. From these groups, only promoters with annotated 5′ UTRs were used in the analysis described in the text.

### Motif identification, co-occurrence and positional enrichment

All strict core promoter motif were identified using meme (parameters: -maxsize 20000000 -p 14 -dna -nmotifs 10 -minw 6 -maxw 15 -mod zoops)^74^. Each motif was searched against the Jaspar CORE and Jaspar POLII motif database^75^ using TomTom (http://meme.ebi.edu.au/meme/tools/tomtom), STAMP (http://www.benoslab.pitt.edu/stamp/) and manual searches and renamed according to their most similar motif in the database or literature, if any. Motif frequency matrixes were retrieved from MEME output, converted into Homer format and used to identify of frequency across the promoter region^76^.

Positional motif enrichment analysis was carried out using Homer^76^. All analyses on the same strand were done using the “-norevopp” parameter. For motif positional enrichment all motifs of interest were searched in the core promoter region of bidirectional, putative bidirectional and unidirectional promoters (see above) using the annotatePeaks.pl script from the Homer pipeline −400 and +100 nts around the TSS in adjacent windows of 10 bp each. All frequency metrics refer to the number of identified motifs per bin divided by the total number of peaks in region. For genome-wide promoter analysis the “tss” option in Homer was used and only sequences that had full regions around the identified motif (no gaps) were analyzed. The dinucleotide frequency of core promoter regions was calculated using the faCount tool from the UCSC utilities^72^, while the overall nucleotide and dinucleotide frequency across the promoter regions was quantified using the annotatePeaks.pl script included in Homer^76^. Core promoters were defined as the region 150 bp upstream and 50 bp downstream of the TSS for genes that had an annotated 5′UTR supported by RNA-seq and EST data^34^.

For the cumulative PAS and 5′SS motif identification, a position specific matrix for the vertebrate PAS consensus (AWUAAA) and the 5′SS consensus (Jaspar database – motif SD0001.1) were searched 500 bp downstream of all TSS or motifs of interest identified in the 400 bp around annotated TSS. The frequencies in adjacent windows of 10 bp were used to calculate the cumulative frequency in the corresponding strand. IUPAC consensus sequences were obtained by converting meme motifs to jaspar format using the meme-suite meme2jaspar followed by prediction of the consensus sequence using the web-version of rsa-tools convert-matrix^77^.

## Additional Information

**How to cite this article**: Fernandez-Valverde, S. L. and Degnan, B. M. Bilaterian-like promoters in the highly compact *Amphimedon queenslandica* genome. *Sci. Rep.* **6**, 22496; doi: 10.1038/srep22496 (2016).

## Supplementary Material

Supplementary Information

Supplementary Dataset 1

Supplementary Dataset 2

Supplementary Dataset 3

## Figures and Tables

**Table 1:** Genome statistics of eukaryote genomes.

	*Sce*	*Cow*	*Sro*	*Mbr*	*Aqu*	*Mle*	*Pba*	*Tra*	*Nve*	*Dme*	*Cin*	*Tru*	*Hsa*
Genome Size (without mitochondria) [Mb]	12.1	28.0	55.4	41.6	166.7	155.9	156.1	105.6	356.6	168.7	115.2	393.3	3,095.7
Genes	7,126	10,123	11,736	9,196	40,122	16,548	19,521	11,520	27,270	26,951	17,289	47,841	22,810
Bases on scaffolds >50 kb [Mb]	12.1	27.4	54.8	40.6	109.9	135.9	41.7	99.3	275.5	168.7	101.8	339.4	3,095.7
Gene Density (Genes/Mb)	590.3	362.0	211.7	220.9	240.7	106.2	125.0	109.1	76.5	159.7	150.1	121.6	7.4
Median Gene Density (Genes/50 kb)	29	16	10	11	9	5	2	6	4	3	7	4	0
Percentage of Gene deserts (50 kb)	0.00	0.18	0.27	1.31	2.73	5.76	16.96	7.16	8.01	30.54	1.69	20.24	80.47
Mean introns per gene	0.1	4.0	7.5	6.6	4.2	4.5	4.2	7.4	4.3	4.9	6.2	12.3	6.3
Mean intron size (bp)	271	157	252	171	326	891	504	283	798	1569	476	659	5923
Number of 50 kb gene depleted regions	0	1	3	11	70	182	195	144	474	1,033	36	1,446	45,248
% AT	61.9	46.2	44.0	45.1	64.2	61.1	57.2	67.3	59.4	58.3	64.3	54.5	59.1

Organism abbreviations: *Sce - Saccharomyces cerevisiae, Cow - Capsaspora owczarzaki, Sro - Salpingoeca rosetta, Monosiga brevicollis, Aqu - Amphimedon queenslandica, Mle - Mnemiopsis leidyi, Pba - Pleurobrachia bachei, Tad - Trichoplax adhaerens, Nve - Nematostella vectensis, Dme - Drosophila melanogaster, Cin - Ciona intestinalis Tru - Takifugu rubripes, and Hsa - Homo sapiens.*

**Table 2:** *Amphimedon queenslandica* gene orientation.

Gene orientation(Number of Promoter regions)	Overlapping	Not overlapping	Not overlapping (<1 kb)	% Regions	% Regions (<1 kb)
Head to head	549	5,652	3,427	22.3%	21.7%
Tail to tail	1,580	4,250	2,190	21.0%	20.6%
Head to tail	1,168	14,627	9,408	56.8%	57.7%
TOTAL	3,297	24,529	15,025	27,826	18,322
