# Supplementary material for: Bilaterian-like promoters in the highly compact Amphimedon queenslandica genome
Source: Sci Rep. 2016 Mar 2;6:22496. doi: 10.1038/srep22496 (PMC4773876; doi:10.1038/srep22496)
Supplement: Supplementary Information [file srep22496-s1.pdf]

# Supplementary Information

## Bilaterian-like promoters in the highly compact *Amphimedon queenslandica* genome

Selene L. Fernandez-Valverde[1,2] and Bernard M. Degnan[1*]

[1] School of Biological Sciences, The University of Queensland, Brisbane 4072, Australia

[2] Present address: Cátedras CONACyT, Laboratorio Nacional de Genómica para la Biodiversidad (LANGEBIO). CINVESTAV, Irapuato, Guanajuato, México.

* Corresponding author

**Email addresses:**

SLFV: sfernandezv@langebio.cinvestav.mx

BMD: b.degnan@uq.edu.au

**Corresponding author contact details:**

Bernard M. Degnan
School of Biological Sciences
University of Queensland
Brisbane QLD 4072 Australia
Phone: +61 7 336 52467
E-mail: b.degnan@uq.edu.au

# Supplementary note

## Promoter motif co-occurrence

To understand how *Amphimedon* core promoter motifs might be acting together to initiate transcription, we surveyed how often the six most prevalent motifs (Figure 3) and the vertebrate TATA–box co-occurred in the either the sense or both strands, and how often they overlapped with each other in *Amphimedon* promoters (Table S3). The Sp1/GC-box motif and the Sp1rc motif are almost twice as likely to co-occur in the same strand and almost three times more likely to co-occur on both sense and antisense strands than expected by chance (Table S3). Surprisingly given their reverse complementarity, only half of these co-occurrences involve overlapping Sp1/GC-box and Sp1rc motifs on opposite strands (Table S3). Meanwhile both Sp1/GC-box and Sp1rc motifs do not co-occur with the TATA-box above what is expected by chance (~25%) (Table S3). Promoters with either an Sp1/GC-box or Sp1rc motif are, on average, 1.7 times more likely to have a YY1/Kozak motif than expected by chance (Table S3), unlike the TATA-box motif (Table S3).

# Supplementary Methods

## Motif co-occurrence analysis

To quantify motif co-occurrence all motifs of interest were searched in the core promoter region using annotatePeaks.pl 400 bp around the TSS and using the –matrix option. For sense strand analysis the "-norevopp" parameter was used, and for non-overlapping motif analyses the "-matrixMinDist 1" parameter was used.

## Supplementary Figures

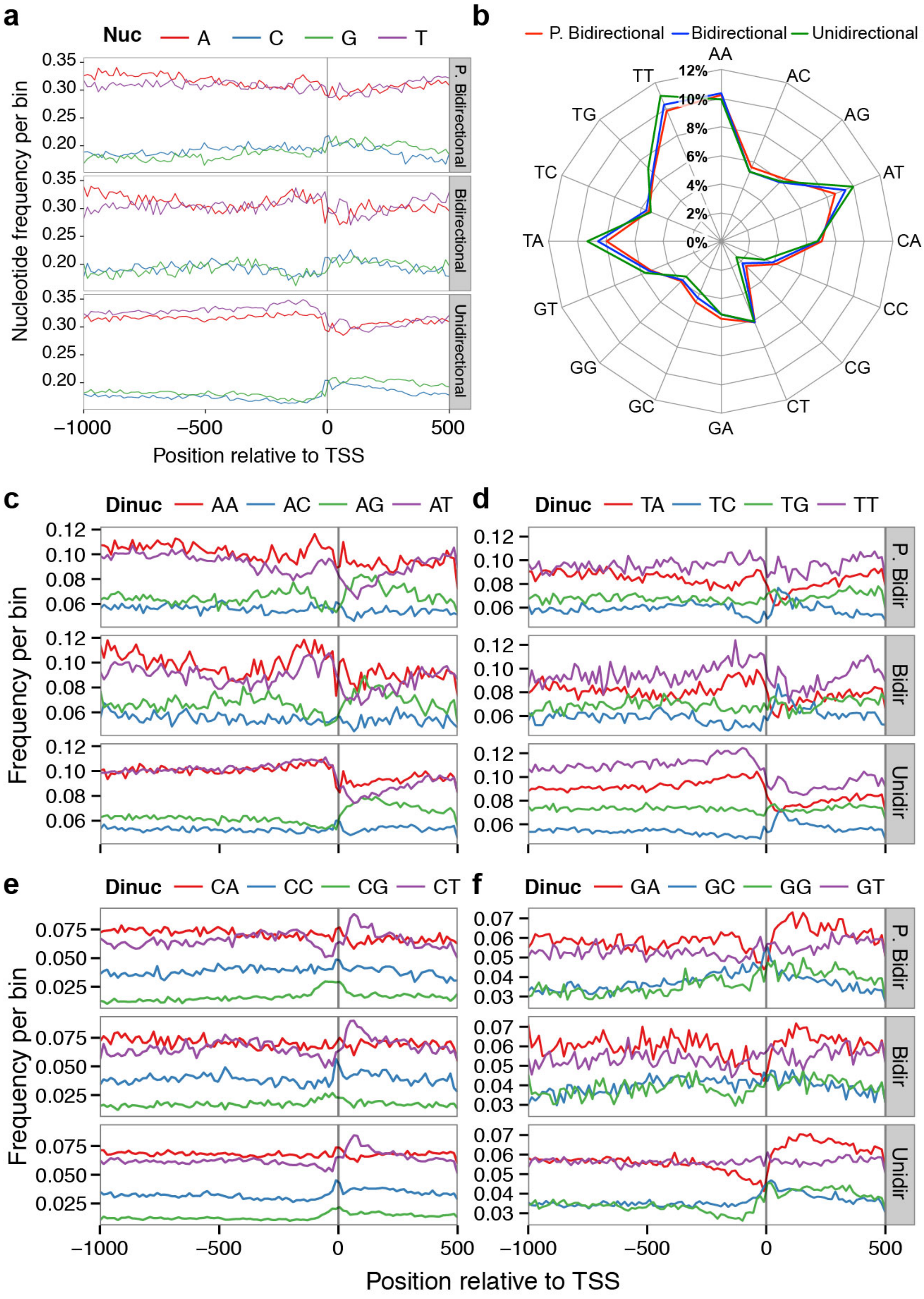


**Supplemental Figure 1 – Mono and dinucleotide composition of *Amphimedon queenslandica* promoters. a**) Frequency of adenine (A), cytosine (C), guanine (G) and thymine (T) per 15 bp bin across -1000 bp and +500 bp from the transcription start site (TSS – grey vertical line) defined as the 5' UTR start of unidirectional (bottom panel), bidirectional (middle panel) and putatively bidirectional (top panel) promoters. **b)** Radar plot showing the dinucleotide percentage in the core promoter region -150 bp and +50 bp off the

5’ UTR start of unidirectional, bidirectional and putatively bidirectional promoters. The distance from the centre of the plot represents the percentage of each dinucleotide (dark radiating lines) according to the vertical scale shown only for dinucleotide AA. Only promoter regions that had no overlap with other regions are displayed. **c-f)** Dinucleotide frequency per 15 bp bin across -1000 bp and +500 bp from the TSS (grey vertical line) defined as the 5’ UTR start of unidirectional (bottom panel), bidirectional (middle panel) and putatively bidirectional (top panel) promoters. The colour-code for each dinucleotide is shown in the legend on top of each panel.

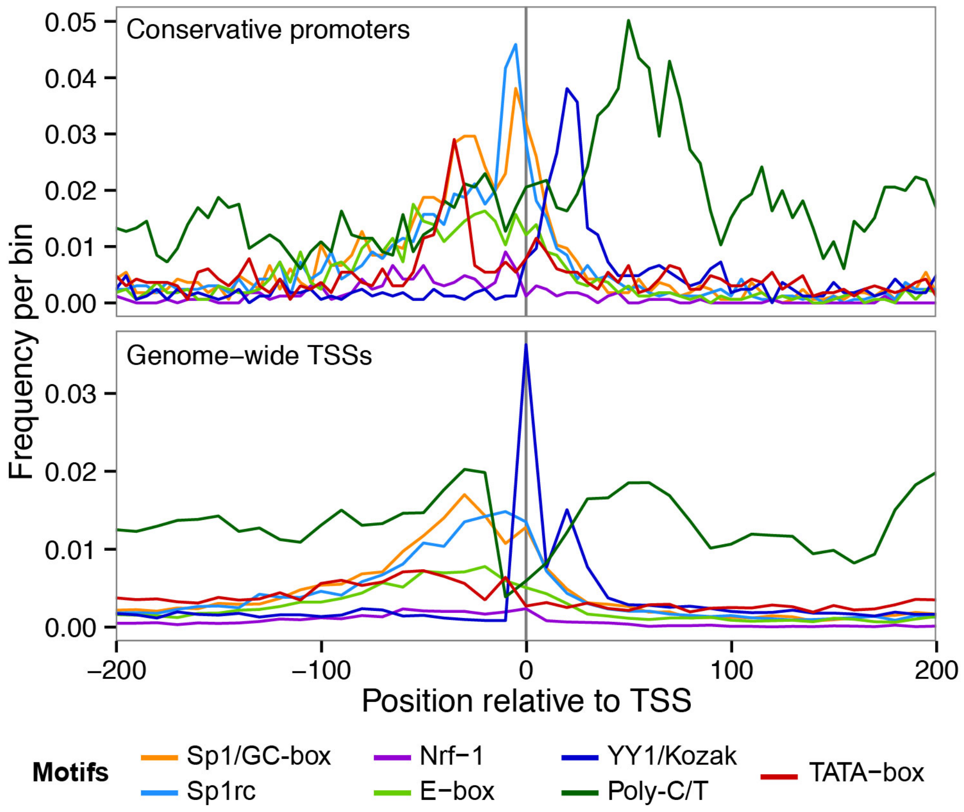


**Supplemental Figure 2 – Core promoter motifs distribution across *Amphimedon queenslandica* promoters.** The frequency per 5 bp bin of the TATA-box (red), Sp1/GC-box (orange), Sp1rc (light blue), Nrf-1 (purple), E-box (bright green), YY1/Kozak (navy blue) and Poly-C/T (dark green) motifs across -200 bp and +200 bp from the TSS (grey vertical line) in conservative (top panel) and genome-wide promoters (bottom panel). In genome-wide promoter regions, the YY1/Kozak sequence overlaps with the TSS as most genes in *Amphimedon* lack an annotated 5' UTR, thus, for most genes, the start of the gene model corresponds to the start of the identified open reading frame (ORF).

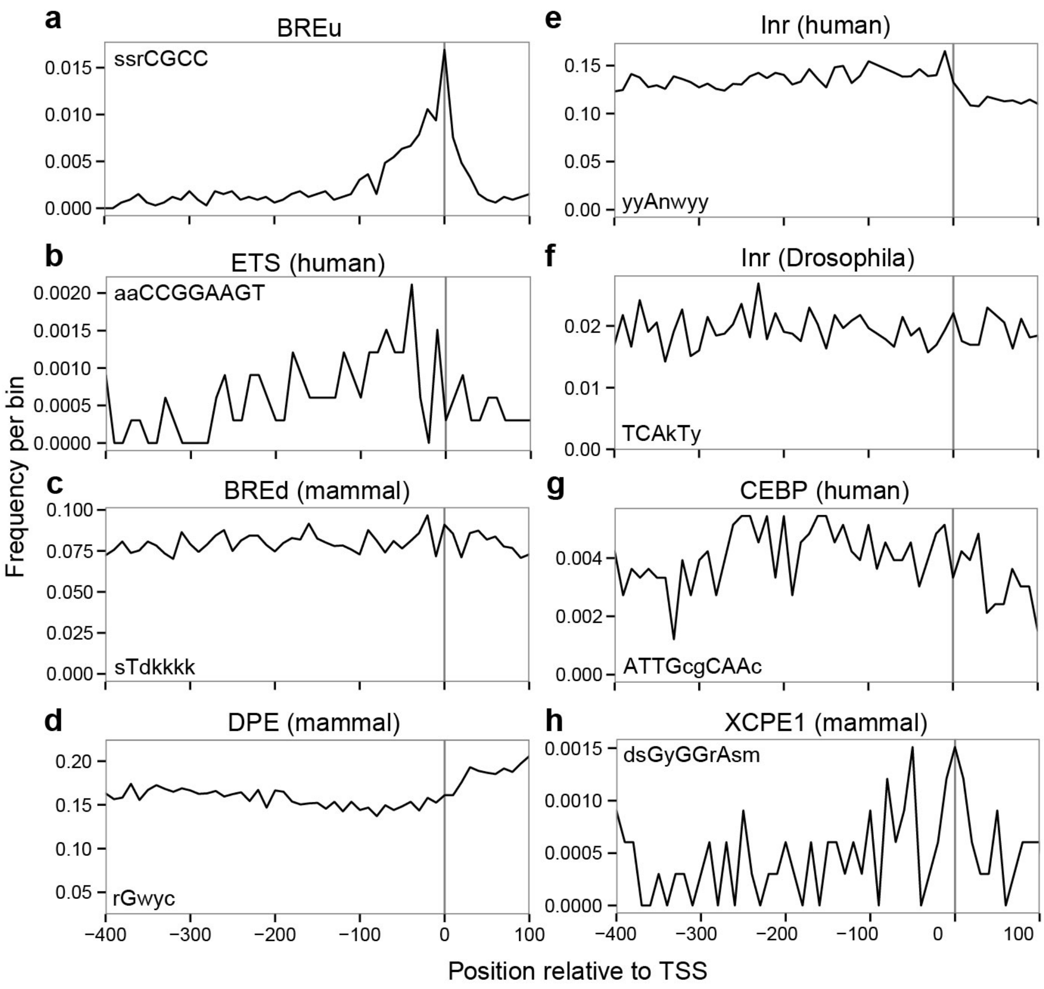


**Supplemental Figure 3 – Distribution of known core promoter motifs across *Amphimedon queenslandica* promoters -** Frequency per 5 bp bin of consensus **a)** upstream B-recognition element (BREu), **b)** Human ETS, **c)** downstream B-recognition element (BREd), **d)** mammalian downstream promoter element (DPE), **e)** human initiator (Inr), **f)** *Drosophila* Inr, **g)** human CEBP, and **h)** mammalian XCPE1 (y-axis) across -400 bp and +100 bp from the TSS (grey vertical line) defined as the 5' UTR start strict promoters (see text). The sequences on the lower-left corner of each plot and upper left corner of panel a, b and h show the consensus motif in IUPAC code.
